# Proximity of the middle meningeal artery and maxillary artery to the mandibular head and mandibular neck as revealed by three-dimensional time-of-flight magnetic resonance angiography

**DOI:** 10.1007/s10006-021-00960-0

**Published:** 2021-05-23

**Authors:** Daphne Schönegg, Raphael Ferrari, Julian Ebner, Michael Blumer, Martin Lanzer, Thomas Gander

**Affiliations:** Department of Oral and Maxillofacial Surgery, University Hospital of Zürich, Frauenklinikstrasse 24, 8091 Zürich, Switzerland

**Keywords:** Maxillary artery, Middle meningeal artery, Mandibular condylar process, Topographic relationship

## Abstract

**Purpose:**

The close topographic relationship between vascular and osseous structures in the condylar and subcondylar region and marked variability in the arterial course has been revealed by both imaging and cadaveric studies. This study aimed to verify the previously published information in a large sample and to determine a safe surgical region.

**Methods:**

We analyzed the three-dimensional time-of-flight magnetic resonance angiography images of 300 individuals.

**Results:**

The mean distance between the middle meningeal artery and the apex of the condyle or the most medial point of the condyle was 18.8 mm (range: 11.2–25.9 mm) or 14.5 mm (range: 8.8–22.9 mm) respectively. The course of the maxillary artery relative to the lateral pterygoid muscle was medial in 45.7% of cases and lateral in 54.3%. An asymmetric course was evident in 66 patients (22%). The mean distance between the maxillary artery and condylar process at the deepest point of the mandibular notch was 6.2 mm in sides exhibiting a medial course (range: 3.7–9.8 mm) and 6.6 mm in sides exhibiting a lateral course (range: 3.9–10.4 mm). The distances were significantly influenced by age, gender, and the course of the maxillary artery.

**Conclusion:**

Our study emphasizes the marked inter- and intra-individual variability of the maxillary and middle meningeal arterial courses. We confirmed the proximity of the arteries to the condylar process. Extensive surgical experience and thorough preparation for each individual case are essential to prevent iatrogenic vascular injury.

## Introduction

The mandibular condylar process is a common surgical site after trauma and when reconstruction is required [1–6]. Surgeons are well-aware that the arteries supplying the temporomandibular joint (TMJ) run close to the facial bones, particularly to the mandibular condylar process. The limited visibility and accessibility associated with minimally invasive and endoscopic procedures in narrow anatomical regions are associated with a risk of iatrogenic vascular injury, which leads to bleeding that is difficult to control and potentially life-threatening.

Several imaging studies have described the close relationships between the maxillary and middle meningeal arteries and osseous structures [7–12]. However, the minimal distances between these structures remain difficult to predict for an individual patient; at present, it is not possible to generally delimit a safe surgical region. This is attributable principally to high inter- and intra-individual variability in the arterial course, which may reflect the complexity of embryological development [13–15]. Two main subtypes of maxillary arterial courses have been described based on their relationship with the lateral pterygoid muscle: a lateral or superficial type and a medial or deep type. The course is thought to be partly dependent on ethnicity and gender [16–18].

Here, we measured the distances between the maxillary and middle meningeal arteries and osseous landmarks, as revealed by three-dimensional time-of-flight magnetic resonance angiography (3D-TOF-MRA). This imaging technique is well suited to visualize vascular structures, especially in neuroradiological assessments. Due to its standard use in stroke diagnostics, a large number of data sets are easily available for further analysis. Compared to other imaging modalities such as contrast-enhanced computed tomography, 3D-TOF-MRA is characterized by good spatial resolution, contrast, and ease of use. We aimed to verify the previously published information in a large sample and to define a safe surgical region. A tabular summary of study results from the past decade complements this study.

## Materials and Methods

A total of 300 individuals, equally distributed by gender (150 women, 150 men), who had undergone 3D-TOF-MRA as part of the routine diagnostic workup of the Swiss Stroke Registry between January 2017 and December 2018 in the Department of Neurology, University Hospital of Zürich, were included in this study.

Data were acquired using either an Ingenia (Philips, Amsterdam, Netherlands) or Magnetom (Siemens, Munich, Germany) 3 T magnetic resonance imaging (MRI) scanner, with the following settings: TR, 20 ms; TE, 3.43 ms; flip angle, 20°; slice thickness, 0.6 mm; and acquisition matrix, 320 × 238.

The inclusion criteria were age ≥ 20 years at the time of imaging, diagnosis of a transient ischemic attack (TIA) or TIA mimic, and consent (i.e., no documented refusal) to the further use of data. The exclusion criteria were osseous changes (caused by prior facial fracture, TMJ surgery, severe degenerative TMJ disease, a tumor, or an anatomical variant such as a bifid condyle) and any vascular pathology (prior carotid dissection, severe arteriosclerotic disease, carotid stenosis, cerebrovascular bypass surgery, or stroke). Patients with poor-quality images (usually because of movement artifacts) were also excluded.

Data on age and gender were extracted from patient charts. The 3D-TOF-MRA scans were collected from our picture archiving and communication system (PACS), imported into iPlan 3.0.5 ENT software (Brainlab, Munich, Germany), and aligned with predefined planes (the Frankfurt horizontal plane, the coronal plane connecting the outer acoustic meatus, and the median sagittal plane along the cerebral falx).

**Fig. 1:**
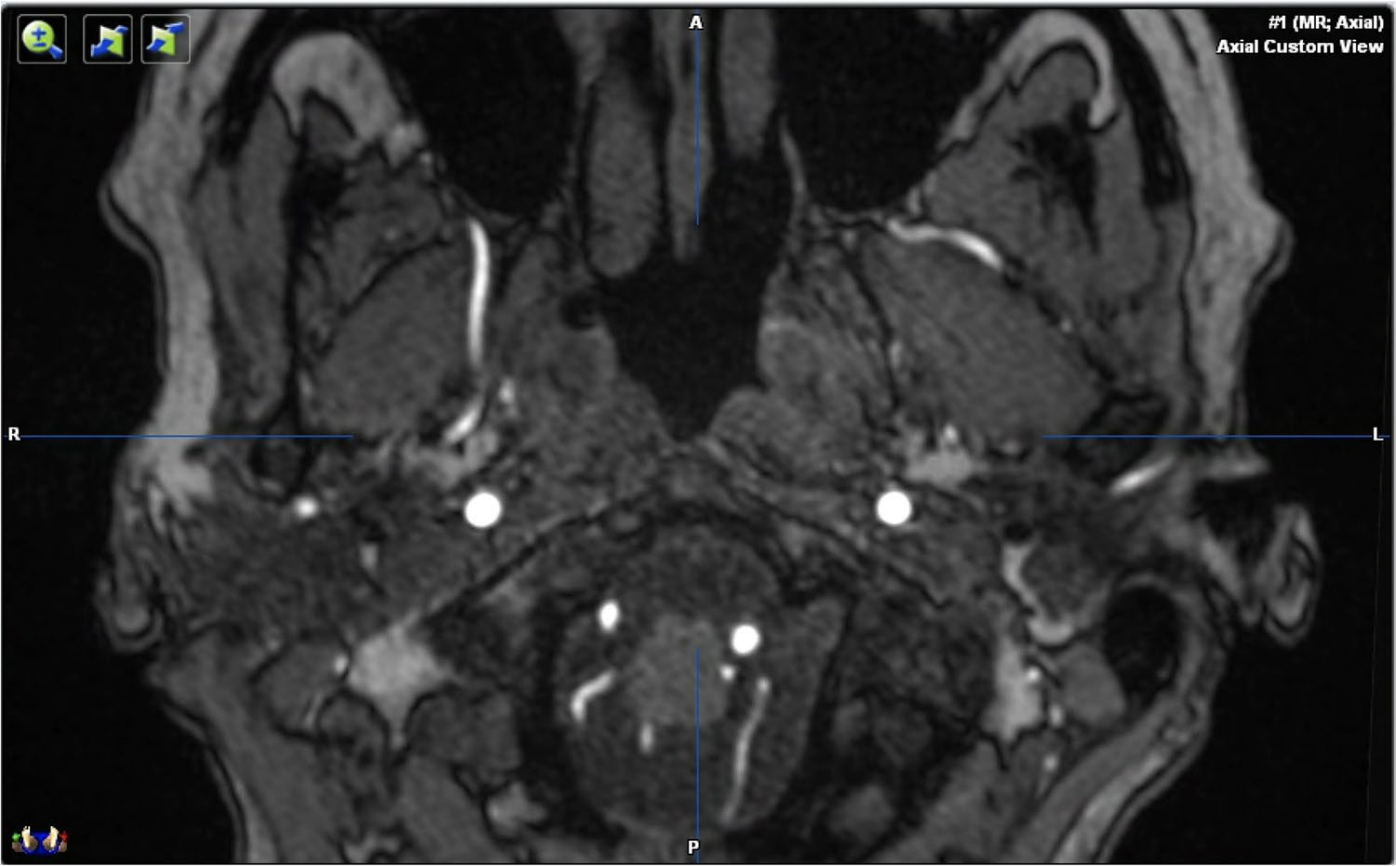
Asymmetric course of the maxillary artery running medial to the lateral pterygoid muscle on the right and lateral to the lateral pterygoid muscle on the left side in the axial plane

**Fig. 2:**
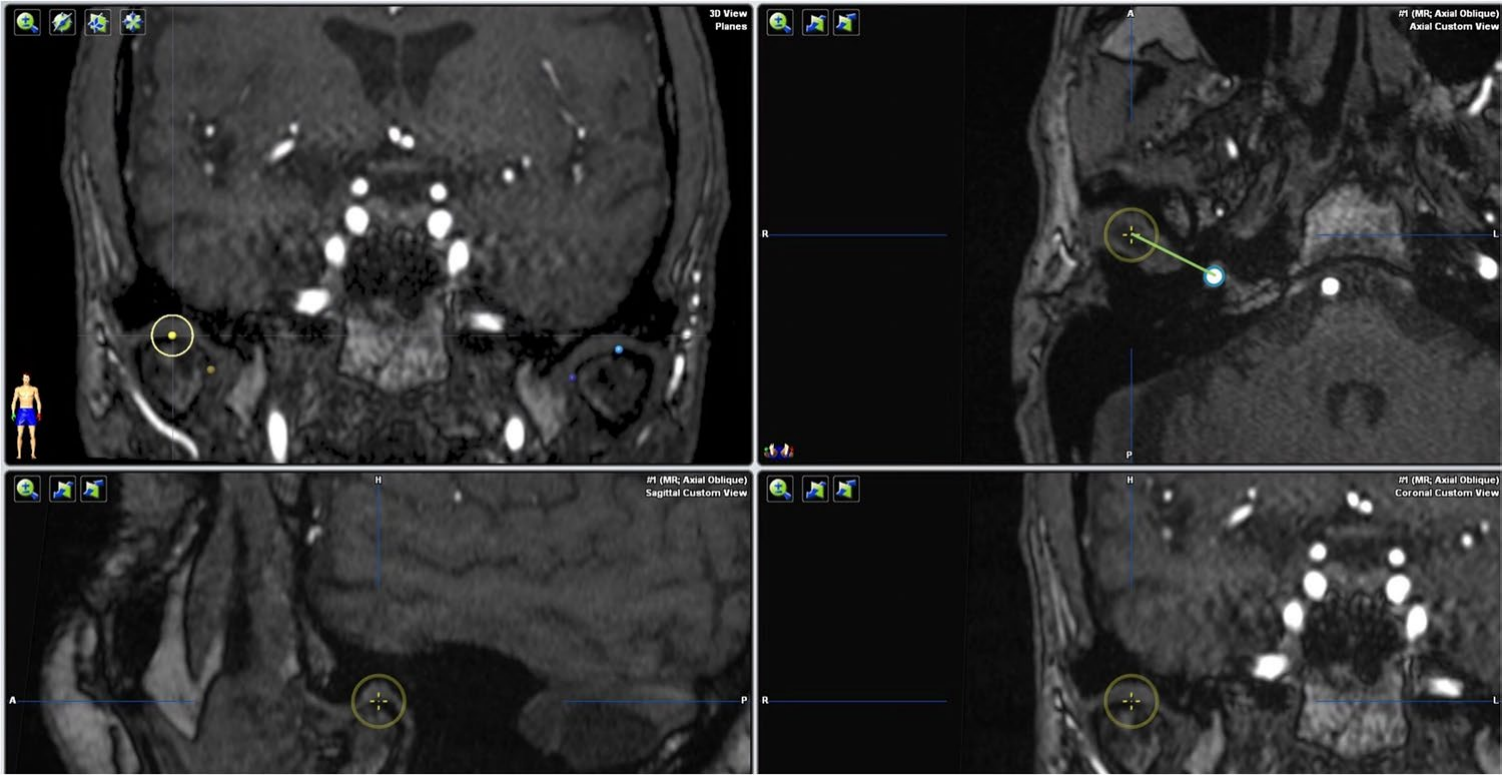
Measurement of the distance between the most apical point of the condyle and the middle meningeal artery in the axial plane

The courses of the right and left maxillary arteries were classified as either medial (deep) or lateral (superficial) by reference to the lateral pterygoid muscle, as shown on axial images (Fig. 1). The distance between the most apical point of the condyle and the middle meningeal artery was measured in the axial plane using iPlan. For this, the most apical point of the mandibular condyle was identified in the coronal and sagittal planes and marked using the iPlan “reference point” tool. Each axial view was automatically re-centered on the reference point and the center of the middle meningeal artery was then marked (Fig. 2).

**Fig. 3:**
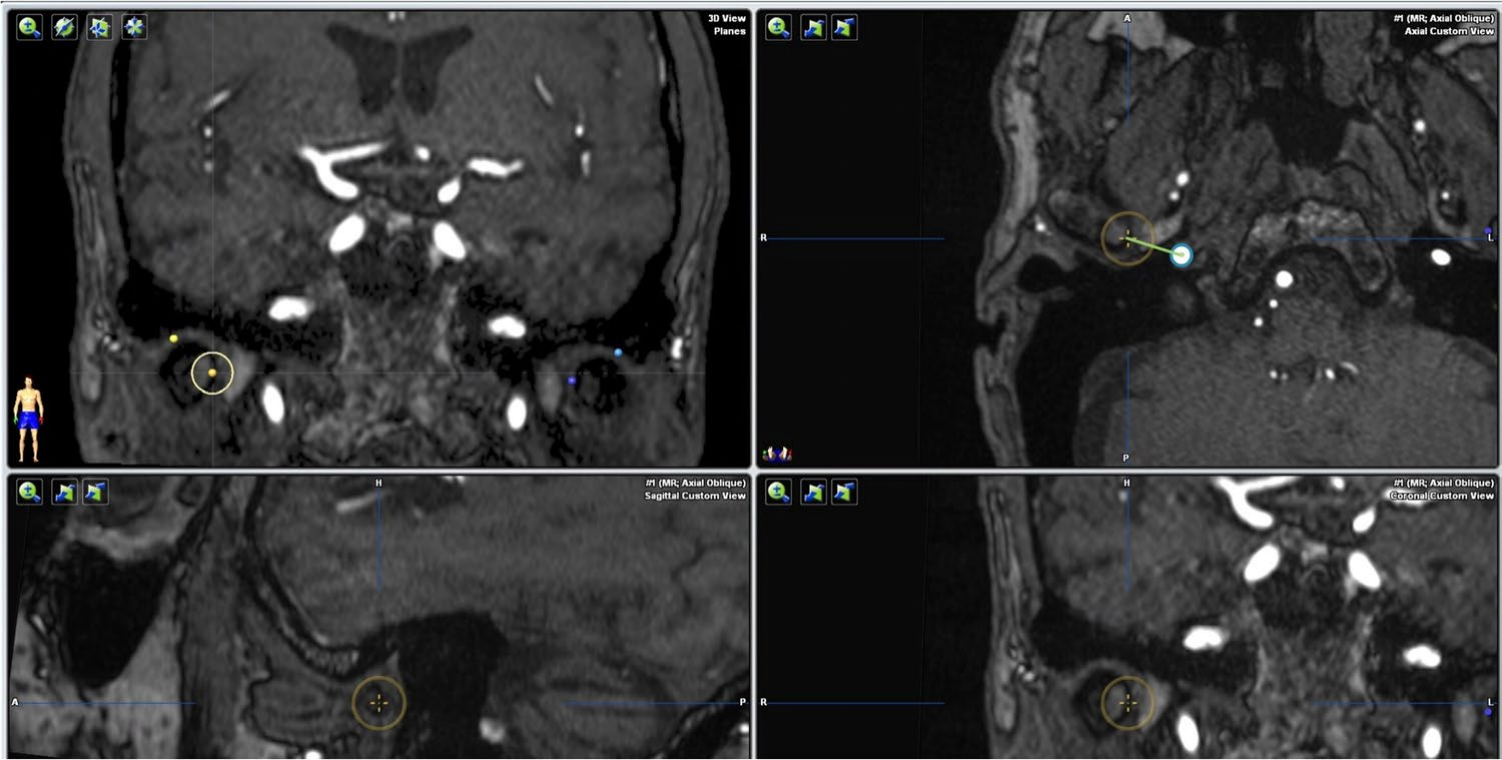
Measurement of the distance between the most medial point of the mandibular condyle and the middle meningeal artery in the axial plane

**Fig. 4:**
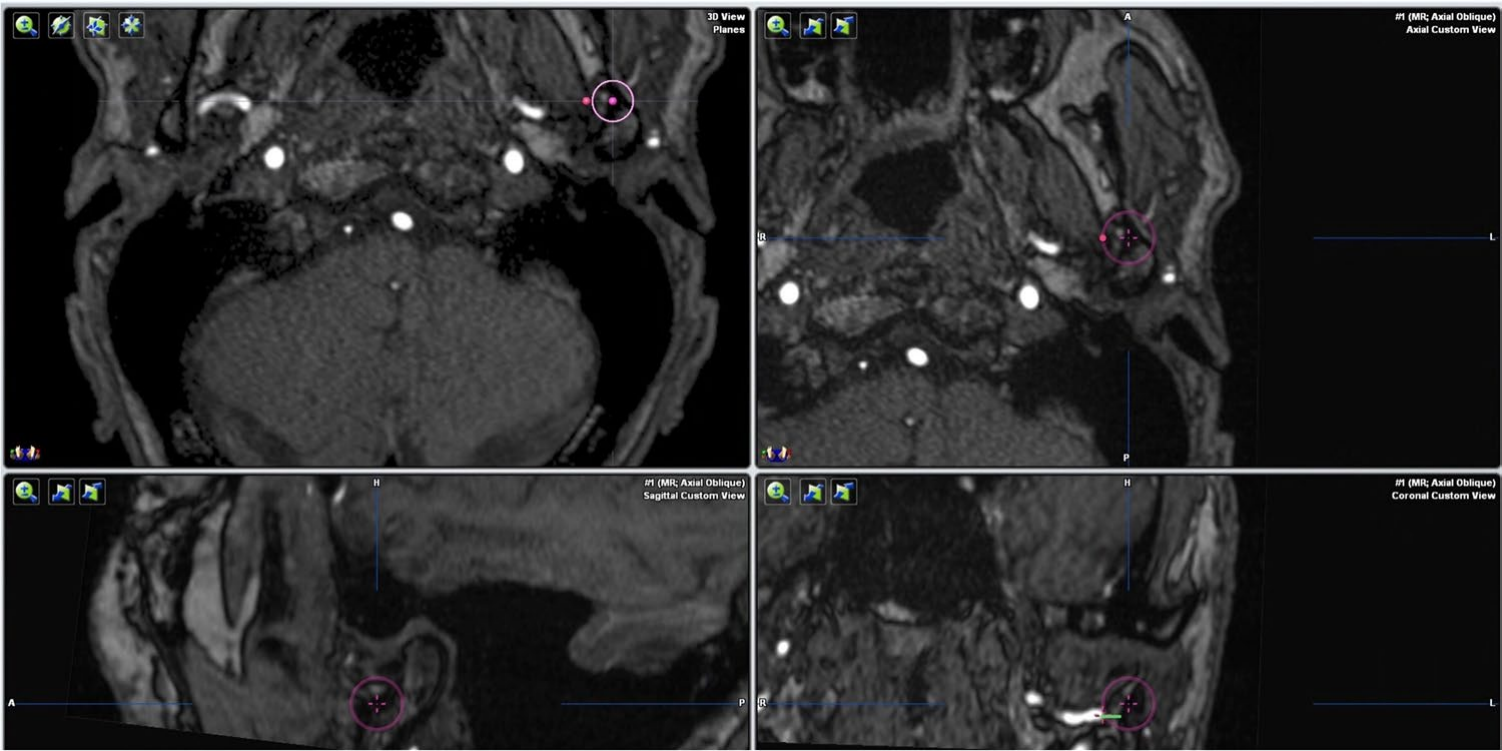
Measurement of the distance between the maxillary artery and the condylar process in the coronal plane

A similar technique was used to measure the distance between the most medial point of the mandibular condyle and the middle meningeal artery. The most medial point of the condyle was identified in the coronal and axial planes. The distance between that point and the center of the middle meningeal artery was then measured on the automatically re-centered axial image (Fig. 3). To measure the distance between the maxillary artery and the condylar process, the deepest point of the mandibular notch was identified and marked in the sagittal plane. The coronal plane was re-centered to run through this reference point, and the most lateral aspect of the maxillary artery was marked. The distance to the outer cortical bone of the condylar process was then measured on a horizontal line parallel to the reference line generated during image alignment (Fig. 4).

All measurements were made on both sides by the same investigator. Statistical analyses were performed using SPSS software (ver. 26.0; IBM Corp., Armonk, NY, USA) and included *t*-tests and Pearson correlation analysis. A *p*-value ≤ 0.05 was considered to indicate statistical significance.

Ethical approval was obtained from the responsible Ethics committee (KEK Zürich; approval no.: 2018–02337). This study thus fulfills the criteria of the Declaration of Helsinki.

## Results

We included 300 patients (150 women, 150 men) with a mean age of 66.1 years (median age, 70.4 years; range: 21.3–94.1 years). The mean age did not differ significantly between the sexes (*p* = 0.311). The course of the maxillary artery relative to the lateral pterygoid muscle was medial on 274 sides (45.7%) and lateral on 326 sides (54.3%). An asymmetric course was evident in 66 individuals (22%; 29 women and 37 men). In 39 of these patients, the right maxillary artery ran medially to the lateral pterygoid muscle.

The mean distance between the most apical point of the condyle and the middle meningeal artery was 18.8 mm (median distance, 18.7 mm; range: 11.2–25.9 mm). The mean distance between the most medial point of the condyle and the middle meningeal artery was 14.5 mm (median distance, 14.4 mm; range: 8.8–22.9 mm). The mean distance between the maxillary artery and outer cortex of the mandibular condyle was 6.4 mm (median distance, 6.2 mm; range: 3.7–10.4 mm); the distance differed significantly between sides with medial and lateral courses relative to the lateral pterygoid muscle (*p* = 0.000). The details are shown in Table 1. All three distances were significantly shorter in women. The distance between the most medial point of the condyle and the middle meningeal artery was significantly smaller on the left than on the right side (*p* = 0.042).

**Table 1:** Subgroup analysis of the mean distances between osseous landmarks and arteries

Distance	Subgroup	Mean (mm)	Median (mm)	*p*-value
From the apex of the condyle to the middle meningeal artery		18.8	18.7	
	Right side	18.9	19.1	0.093
	Left side	18.6	18.3	
	Medial type	18.8	18.8	0.626
	Lateral type	18.7	18.6	
	Men	19.5	19.7	0.000*
	Women	18.1	18.1	
From the most medial point of the condyle to the middle meningeal artery		14.5	14.4	
	Right side	14.7	14.6	0.042*
	Left side	14.4	14.2	
	Medial type	14.6	14.4	0.982
	Lateral type	14.5	14.4	
	Men	14.8	14.6	0.004*
	Women	14.3	14.1	
From the condylar process to the maxillary artery		6.4	6.2	
	Right side	6.4	6.2	0.956
	Left side	6.4	6.4	
	Medial type	6.2	5.9	0.000*
	Lateral type	6.6	6.6	
	Men	6.6	6.5	0.000*
	Women	6.2	6.0	

^*^Significant difference at *p* ≤ 0.05

Pearson correlation analysis revealed a significant linear correlation between measurements on the left and right sides for all three distances; the correlation coefficients were 0.635 for the maxillary arterial measurements, and 0.772 and 0.808, respectively, for measurements involving the most apical and most medial condylar aspects (all *p* = 0.000). The mean distances between the middle meningeal artery and the most apical and most medial aspects of the condylar head were significantly correlated (0.669; *p* = 0.000). Age at the time of imaging was significantly correlated with the distances between the middle meningeal artery and the most apical and most medial condylar points (0.249 and 0.361; both *p* = 0.000), respectively.

## Discussion

By using the Swiss Stroke Registry, we could include more patients in this study than in previous studies and achieve an equal sex distribution. We applied strict inclusion and exclusion criteria to minimize confounding. The minimum age of the patients was 20 years, to minimize any effect of residual mandibular growth. We lacked data on the extent of mouth-opening during MRA [7] and occlusion type [19, 20], both factors that can influence the position of the mandible in relation to the skull base. Our patients were older than those of previous studies (mean age, 66 years). This may be relevant in that degenerative TMJ disease or tooth loss, which affects occlusion and condylar morphology, is more common in the elderly [21–23].

Measurements were made on 3D-TOF-MRA images, which reveal arteries and bones at high spatial resolution, with a good signal-to-noise ratio and possibly higher accuracy due to the capture of dynamic flows. iPlan ENT software allows for automatic alignment, re-centering, and measurement of distances, thus enabling reproducible, reliable, and rapid analysis.

The maxillary and middle meningeal arteries are especially prone to iatrogenic injury during osteotomy, fragment repositioning, screw insertion, or surgical dissection near the articular eminence (prior to insertion of a TMJ prosthesis), especially in patients with TMJ ankylosis [24–30]. The reference points selected for our new measurement technique are chosen to correspond to landmarks that are easily identifiable intraoperatively. They can thus serve as a guide structure during surgery and facilitate surgeon orientation when avoiding proximity to the vessels.

The shape of the condylar process is variable and is influenced by several factors including age and gender [31]. Thus, we used the most apical and medial condylar points as references. Both points are clearly identifiable regardless of condylar shape and volume. The center of the middle meningeal artery was also a reference point, such that variation in vessel diameter [12, 32, 33] and branching pattern [34–36] were not factors. The distance between the maxillary artery and condylar process was measured in the plane running through the deepest point of the mandibular notch, because that plane is easy to identify. We measured the distance from the outer cortex of the condylar process to the most lateral point of the maxillary artery, to balance the variation in bone thickness against the tortuosity of the artery and thus ensure consistent cuts.

The course of the maxillary artery relative to the lateral pterygoid muscle has been examined in several studies (Table 2). Of our patients, 22% exhibited an asymmetric course, in agreement with the study of Gulses et al. [17], in which 21% of 209 patients showed an asymmetric course, but far higher than in studies with smaller numbers of cases (see Table 2). Wang et al. [18] comprehensively summarized the prevalence rates of lateral and medial courses reported by various studies. We found five additional relevant publications (Table 3). Several authors reported that the lateral course was more common in Asian and Black populations. The Swiss Stroke Registry does not document ethnicity. We noted a medial course in 45.7% of sides, comparable to the proportion reported in other European studies.

**Table 2:** Literature review: prevalence of symmetric and asymmetric courses of the maxillary artery relative to the lateral pterygoid muscle

	Symmetric (number of patients)	Asymmetric (number of patients, % asymmetric)
First author, year	Balcioglu, 2010 [25] (17) Hussain, 2008 [37] (44) Isolan, 2007 [38] (8) Joo, 2013 [39] (10) Polev, 2019 [40] (6) Uysal, 2011 [41] (7)	Alvernia, 2017 (1/20 = 5%) [42] Arimoto, 2015 (1/19 = 5.3%) [7] Dennison, 2009 (2/53 = 3.8%) [43] Gulses, 2012 (44/209 = 21%) [17] Hwang, 2014 (12/100 = 12%) [10] Maeda, 2012 (n.a.) [44] Orbay, 2007 (1/8 = 12.5%) [28] Otake, 2011 (1/14 = 7.1%) [45]

**Table 3:** Literature review: medial and lateral courses of the maxillary artery relative to the lateral pterygoid muscle

First author, year	Number of sides	Country	Lateral course	Medial course
Isolan, 2007 [38]	16	USA	100%	-
Balcioglu, 2010 [25]	34	Turkey	100%	-
Joo, 2013 [39]	20	Korea	80%	20%
Alvernia, 2017 [42]	20	USA	47.5%	52.5%
Polev, 2019 [40]	12	Russia	83.3%	16.7%

We found that the distances between the middle meningeal artery and the most apical and most medial points of the condyle process were highly variable, ranging from 8.8 to 22.9 mm and 11.2 to 25.9 mm, respectively. These differences of around 10 mm in a narrow anatomical region underline the significant variation, and thus the importance of thorough preoperative preparation.

The course of the maxillary artery is known to be variable and tortuous [10, 14, 46]. Several authors have measured the distances between the maxillary artery and bony reference points. Four studies used methods similar to ours (Table 4). Compared to those studies, our distances were significantly shorter for sides exhibiting a medial maxillary arterial course. The differences are probably attributable to the use of different imaging modalities and alignment methods, and to differences in reference points, measurement techniques, and patient characteristics.

**Table 4:** Literature review: distances between osseous landmarks and the maxillary artery

First author, year	Number of sides	Measurement	Distance (mm)
Nastro Siniscalchi, 2016 [47]	n.a	From the neck of the condyle to the maxillary artery	6.8
Arimoto, 2015 [7]	38	From the mandibular notch to the maxillary artery (measured on MRI scans obtained with the mouth open and closed)	Open: 1.8 ± 0.5 Closed: 1.5 ± 0.5
Hwang, 2014 [10]	200	From the mandibular notch to the maxillary artery (measured on computer tomography scans)	Lateral: 3.6 ± 1.0 Medial: 16.3 ± 3.7
Balcioglu, 2010 [25]	34	From the mandibular notch to the maxillary artery (cadaver measurements) From the medial cortex to the maxillary artery (cadaver measurements)	2.94 ± 0.52 5.38 ± 2.47
Orbay, 2007 [28]	16	From the mandibular notch to the maxillary artery (cadaver measurements)	5.1

The distance between the mandibular condyle and the middle meningeal artery increases significantly with age. This may be attributable to degeneration (condylar flattening) or relaxation of the masticatory muscles, which increases the distance between the skull base and mandible. All three distances were significantly greater in men than women, in accordance with other studies [43, 48–50]. We found that the distance between the most medial point of the condyle and the middle meningeal artery differed significantly between the right and left sides, perhaps attributable to the combined effects of variation in the arterial course and condylar shape.

## Conclusion

The courses of the middle meningeal and maxillary arteries are influenced by age, gender, and ethnicity, and are highly variable and tortuous in all three dimensions. Prediction of individual courses remains difficult. Identification of patients at risk for critical vessel-bone relationships is a first step towards safer surgery. Female gender, a medial course of the maxillary artery relative to the lateral pterygoid muscle, and small cephalometric measurements were risk factors for the proximity of the arteries to the facial bones. A detailed classification of the vascular course, consideration of occlusion type, and analysis of condylar shape would facilitate the interpretation of images. A safe surgical zone around the mandibular condylar process cannot be generally defined; instead, it should be emphasized that the arteries and condyle may be very close, i.e., separated by a distance as small as 3.7 mm. Surgeons must consider the marked inter- and intra-individual variation, and the close relationships between osseous and vascular structures. If an aberrant vascular or bony anatomy is suspected, preoperative visualization can be helpful; this is especially important when planning TMJ prosthesis placement in a patient with ankylosis, because landmarks may be difficult to identify intraoperatively. 3D-TOF-MRA appears to be a suitable imaging modality in these cases. All available measures should be taken to reduce the risk of an intraoperative major vascular injury and bleeding. In addition to preoperative imaging, these include, for example, the use of an intraoperative navigation system, available intraoperative embolization by interventional radiology, or application of vessel loops to the external carotid artery.
